# A Novel Role for Minimal Introns: Routing mRNAs to the Cytosol

**DOI:** 10.1371/journal.pone.0010144

**Published:** 2010-04-12

**Authors:** Jiang Zhu, Fuhong He, Dapeng Wang, Kan Liu, Dawei Huang, Jingfa Xiao, Jiayan Wu, Songnian Hu, Jun Yu

**Affiliations:** 1 CAS Key Laboratory of Genome Sciences and Information, Beijing Institute of Genomics, Chinese Academy of Sciences, Beijing, China; 2 Graduate University of Chinese Academy of Sciences, Beijing, China; University of Georgia, United States of America

## Abstract

**Background:**

Introns and their splicing are tightly coupled with the subsequent mRNA maturation steps, especially nucleocytoplasmic export. A remarkable fraction of vertebrate introns have a minimal size of about 100 bp, while majority of introns expand to several kilobases even megabases in length.

**Principal Findings:**

We carried out analyses on the evolution and function of minimal introns (50–150 bp) in human and mouse genomes. We found that minimal introns are conserved in terms of both length and sequence. They are preferentially located toward 3′ end of mRNA and non-randomly distributed among chromosomes. Both the evolutionary conservation and non-random distribution are indicative of biological relevance. We showed that genes with minimal introns have higher abundance, larger size, and tend to be universally expressed as compared to genes with only large introns and intron-less genes. Genes with minimal introns replicate earlier and preferentially reside in the vicinities of open chromatin, suggesting their unique nuclear position and potential relevance to the regulation of gene expression and transcript export.

**Conclusions:**

Based on these observations, we proposed a nuclear-export routing model, where minimal introns play a regulatory role in selectively exporting the highly abundant and large housekeeping genes that reside at the surface of chromatin territories, and thus preventing entanglement with other genes located at the interior locations.

## Introduction

Gene expression program, rather than a simple assembly line to process mRNAs, is a complex network systematically coordinating many cellular pathways including transcription initiation-elongation-termination, RNA processing, transcription-coupled DNA repair, nuclear export of mRNAs, translation and RNA/protein degradation [1], [2], [3]. In concomitance with transcription and pre-mRNA processing, a dynamic repertoire of proteins are recruited to package mRNA forming the messenger ribonucleoprotein particle (mRNP). The interactions among the protein components of mRNP and other expression machineries can enhance or reduce the rate/efficiency of the coupled reactions, constituting a complex network of co-/post-transcription regulation [4], [5]. The architectural organization of nucleus also provides another level of expression control. Nuclear positions of chromosomes, gene loci and specific genome regions, as well as the spatial interactions among them play important roles in transcriptional regulation [6], [7], [8]. Therefore, expression regulation involves not only the binding of site-specific transcription factors/cofactors but extensive coupling and coordinating among relevant machineries and processes. All of these events are spatially and temporally integrated within the nucleus.

An intriguing example of the coupling among the expression machineries is the observation that RNA splicing influences many subsequent steps of mRNA metabolism such as nucleocytoplasmic export [9]. It was reported that the efficiency of mRNA export can be enhanced 6- to 10-fold for spliced mRNAs relative to their cDNA counterparts in mammalian cells [10]. The current working model for the splicing-dependent nuclear export proposes that the TREX (transcription/export) complex, containing key export factors Aly and UAP56, colocalizes with the splicing machinery in the nuclear speckles. It is recruited to mRNA as a component of the exon junction complex (EJC) at ∼20 bp upstream of the exon-exon junction during splicing. Aly binds to the mRNA export receptor Tap∶p15 heterodimer that interacts with the FG nucleoporins in the pore channel to move the mRNP through the nuclear pore [11], [12], [13]. The magnitude of the splicing-induced enhancement appears to vary from gene to gene [10], and may depend on certain genomic parameters, such as the length and position of introns in the unprocessed transcript. However, the observation that TREX complex can be recruited to cDNA transcripts, although less efficient, implies that splicing can enhance, but is not obligatory, for mRNA export [10]. For naturally intron-less transcripts, export factors were proposed to be recruited by co-transcriptional mechanism or through some specific sequence elements [11]. These results demonstrate that whether a gene has introns and where the introns are have significant influence on gene's nuclear export.

We previously reported a conspicuous feature of vertebrate introns that a remarkable fraction of introns have a lineage-specific minimal size (∼100 bp), which were termed as “minimal introns” [14]. Based on a sequence variation study on human populations and the primate lineage, we proposed that these minimal introns are not “junk” DNA, but may have potential roles in regulating the export of spliced mRNAs from nucleus [14]. In this study, we further analyzed minimal introns in human and mouse genomes. We showed that minimal introns are evolutionarily conserved in terms of both length and sequence as compared to large introns. Minimal introns preferentially locate toward 3′ end of mRNA and are non-randomly distributed among chromosomes. Both the evolutionary conservation and non-random distribution indicate their biological relevance.

In order to understand their functions, we analyzed genes with minimal introns and found some unique characteristics associated with the presence of minimal introns. In general, genes with minimal introns have higher abundance and larger size, and tend to be universally expressed as compared to genes with only large introns and intron-less genes. The presence of minimal intron is also correlated with the replication timing and chromatin structure of the gene locus, implying specific nuclear positions of these genes. Based on these observations, we proposed a nuclear export routing model where minimal introns play regulatory role to selectively export some highly abundant and large housekeeping genes that reside at the surface of chromatin territory, thus preventing the entanglement with other genes located at the interior locations. Although this model is largely descriptive and hypothetical, it provides a necessary framework to design experiments to test the exact role of minimal introns in post-transcriptional regulation.

## Results

### Minimal introns are evolutionarily conserved

**Figure 1:**
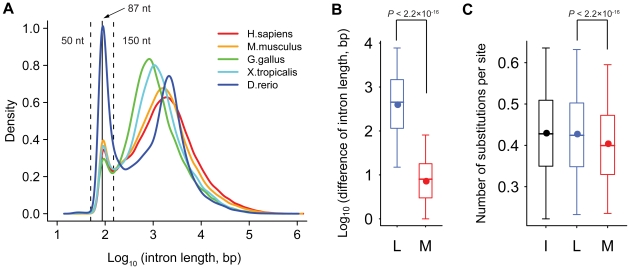
Conservation of minimal introns between human and mouse. The length distributions of vertebrate introns are plotted (A), and minimal introns are defined as those having a length range of 50–150 bp (within the dashed lines). The length differences between human-mouse orthologous introns are significantly larger among large introns (L) and less so among minimal introns (M; B). The number of substitutions per site among minimal introns (M) is significantly lower than that among intronic sequences from large intron (L) and intergenic sequences (I; C). Introns are identified based on the alignments of RefSeq transcripts; and the number of substitutions per site is calculated using BASEML program in PAML package. The boxes depict data between the 25th and 75th percentiles with central horizontal lines and solid circles representing the median and mean values, respectively, and whiskers showing 5th and 95th percentiles.

A remarkable fraction of vertebrate introns have a minimal size (∼100 bp) peaking at the low end of the size distribution, while majority of introns expand to several kilobases even megabases in length (Figure 1A). Over large evolutionary timescale, intron loss and gain have frequently occurred, limiting the identification of orthologous intron [15], [16]. In this study we chose human and mouse for further analysis. They were diverged about 100 million years ago, and intron position relative to coding sequence was proposed to be nearly constant [15]. We can use this correspondence of intron position to identify orthologous introns and monitor intron dynamics.

We analyzed 18,468 human and 18,889 mouse RefSeq loci, containing 175,723 and 165,351 introns, respectively. We defined introns with length of 50–150 bp as minimal introns and those with length of >150 bp as large introns (Figure 1A). In total, 9.4% of human and 10.5% of mouse introns are minimal introns, and 34.3% of human and 33.8% of mouse genes bear minimal introns. From 13,382 human-mouse orthologs, we identified 74,615 reliable human-mouse orthologous intron pairs as those from orthologous genes and having the same position relative to the two coding sequences. These subsets of introns have very similar length distribution as total introns in the two species (data not shown). We observed that 97.1% of introns remain to be either minimal introns or large introns, and exchanging between the two classes occurs rarely (2.9%).

In order to understand the intron evolution between human and mouse, we first analyzed the length differences of human-mouse orthologous introns as a function of intron length. We found that most of large introns fluctuate in length, with a median length difference of 452 bp. In contrast, the lengths of minimal introns are highly conserved, with a median length difference of 8 bp (Figure 1B). At present, the molecular mechanisms that cause intron lengthening/shortening remain poorly understood. Transposon insertion/deletion may be one of the primary reasons, while the fixation of highly conserved sequences was also reported to contribute to intron length dynamics [17]. Moreover, we observed enhanced sequence conservation in minimal introns as compared to intronic sequences from large introns and intergenic sequences (Figure 1C). The number of substitutions per site in minimal introns (with a mean of 0.40) is significantly lower than that in intronic sequences from large introns and intergenic sequences (with means of 0.43 both; Wilcoxon rank sum test, *P*<2.2×10^−16^). We concluded that minimal introns are evolutionarily conserved in terms of both length and sequence.

### Minimal introns reside preferentially toward 3′ end of genes

**Figure 2:**
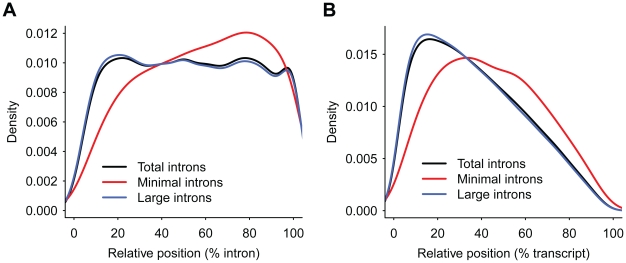
Position preference of minimal introns within genes. Intron position relative to gene structure (A) and transcript (B) are compared between minimal and large introns. Relative to the exon-intron structure, the *i*th intron in a locus with *N* introns is indexed with a position value of *i*/*N* (A). Minimal introns are preferentially located toward 3′ end of the genes as opposite to large introns.

Introns in 5′ UTR are often very large due to higher load of regulatory elements, and introns rarely locate in 3′ UTR because, in most cases, this pattern triggers nonsense-mediated mRNA decay (NMD) pathway [18]. Except for these documented exceptions, we reasoned that if ancient introns randomly lengthened during evolution and minimal introns are reserved by chance, minimal introns would be evenly distributed relative to the exon-intron structure. However, the data denied this expectation. We indexed the *i*th intron in a locus with *N* introns with a position value of *i*/*N*, and observed that minimal introns preferentially reside toward the 3′ end within the sequential arrangement of exon-intron structure (Figure 2A). It's interesting that minimal introns are excluded to be the last intron (with position value of 100%). We also analyzed the intron position relative to the transcript. Because 3′ most exon are generally very large (data not shown), introns in general reside toward 5′ end of the transcript. However, for minimal intron, we also observed a preference toward 3′ end of the transcript (Figure 2B).

### Minimal introns are non-randomly distributed among chromosomes

**Figure 3:**
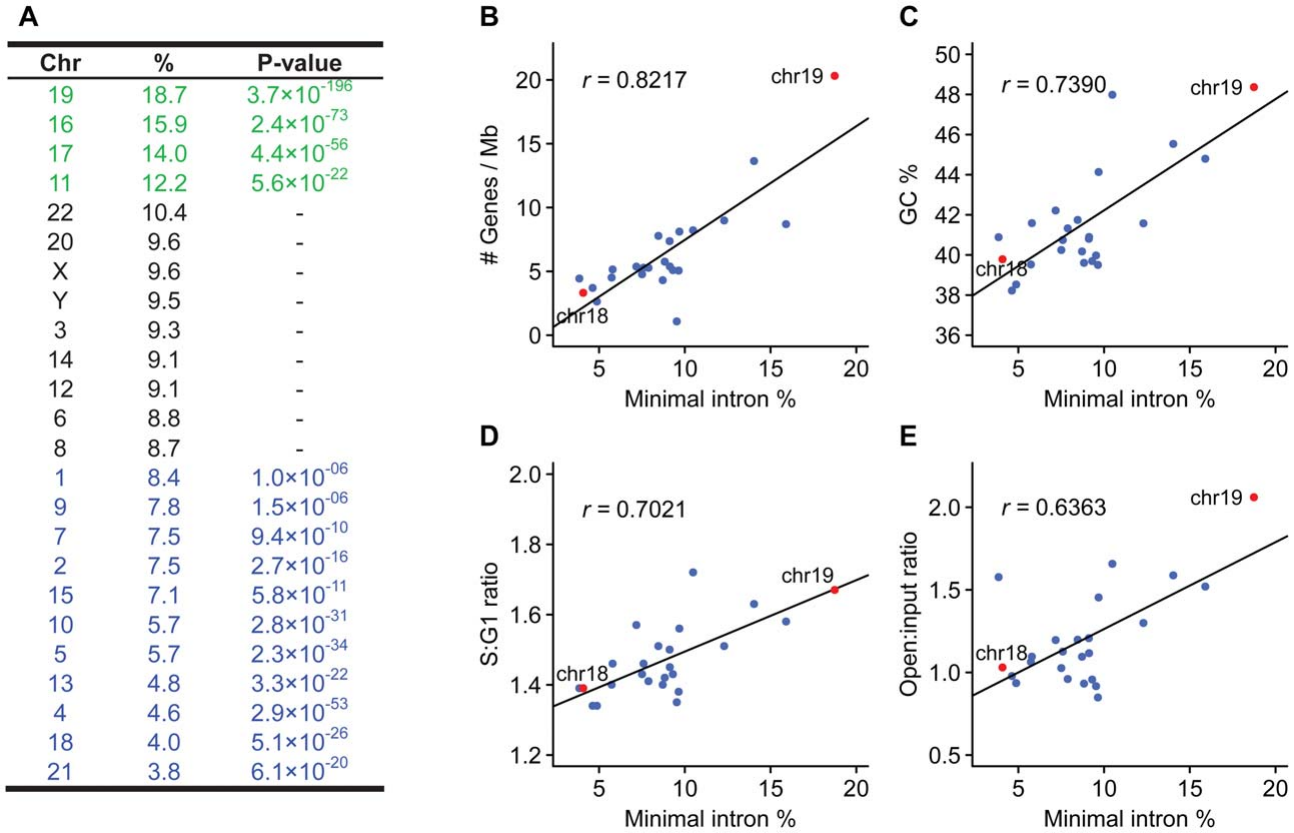
Correlations between chromosome features and minimal intron enrichment. The fraction of minimal intron within a chromosome is used to index its enrichment, and *P*-values are calculated according to the hypergeometric distribution (A). Some chromosomes (green text) are significantly enriched, whereas others (blue text) are significantly deficient. Non-significant *P*-values are omitted (black text). Minimal intron enrichment correlates with gene density (B), GC content (C), replicating time (D) and openness of chromatin structure (E). Replicating time is indexed by S∶G1 ratio, with the larger value representing earlier replication, and chromatin status is demonstrated by open∶input ratio, with the larger value representing the more open chromatin structure [20], [21]. Minimal-intron enriched chromosomes tend to be gene-rich, have higher GC content, replicate earlier and have more open chromatin structure. Chromosome 18 and 19 are colored in red for extreme cases (See main text for details).

We observed that minimal introns are non-randomly distributed among chromosomes. Using the fraction of minimal intron within a chromosome to index its enrichment, some chromosomes (19, 16, 17, 11) are significantly enriched by minimal introns whereas others (21, 18, 4, 13, 5, 10, 15, 2, 7, 9, 1) are significantly deficient (Figure 3A).

It has been reported that chromosome features such as gene density, GC content, replication time, compactness and nuclear position are all correlated—open chromatins tend to locate at the nuclear interior, replicate early, and have higher gene density and GC content as opposite to close chromatins—although the causal relationship among them remains to be elucidated [19], [20], [21]. We observed that minimal intron enrichment is also associated with these chromosome features. Minimal intron enriched chromosomes tend to be gene dense (Figure 3B; *r* = 0.8217, *P* = 8.5×10^−7^), GC-rich (Figure 3C; *r* = 0.7390, *P* = 3.7×10^−5^), replicate earlier (Figure 3D; *r* = 0.7021, *P* = 1.3×10^−4^) and have more open chromatin (Figure 3E; *r* = 0.6363, *P* = 8.3×10^−4^).

Two striking example are chromosome 19 and 18 (Figure 3). Although having similar DNA content (64 Mb and 76 Mb, respectively), they differ significantly in their gene and GC content. Chromosome 19 is gene-dense (20.3 genes/Mb) and has high GC content (48.4%), whereas chromosome 18 is gene-poor (3.3 genes/Mb) and has low GC content (39.8%). Chromosome 19 is the most minimal intron-enriched chromosome (with 18.7% of total introns, *P* = 3.7×10^−196^), but chromosome 18 is among the most minimal intron deficient (with 4.0% of total introns, *P* = 5.1×10^−26^). Chromosome 19 replicates early and has open chromatin structure (with a mean S∶G1 and open∶input ratio of 1.67 and 2.06, respectively) as compared to chromosome 18 (with a mean S∶G1 and open∶input ratio of 1.39 and 1.03, respectively). In human lymphocyte nuclei, which exhibit an spherical shape, chromosome 19 is consistently localized toward the nuclear center without any detectable attachment to the nuclear envelope, whereas chromosome 18 is positioned close to the nuclear border [22], and this nuclear arrangement was reported to be highly conserved [23].

### Genes with minimal introns have unique characteristics

The evolutionary conservation, 3′-positional preference and non-random chromosomal distribution all indicate that minimal introns may have special biological functions. We previously showed that minimal introns are not randomly distributed among genes [14]. Therefore, it is expected to observe their functional relevance from the characteristics of genes with minimal introns. We carried out gene ontology (GO) analysis on the functional annotation of 6,327 (34.3%) genes with minimal introns (Table S1), and found several significantly overrepresented functional groups (Figure S1). Genes related to the cellular structure are among the most enriched functional groups, such as nuclear envelope and cytoskeleton. Most of these genes are related to various housekeeping functions.

**Figure 4:**
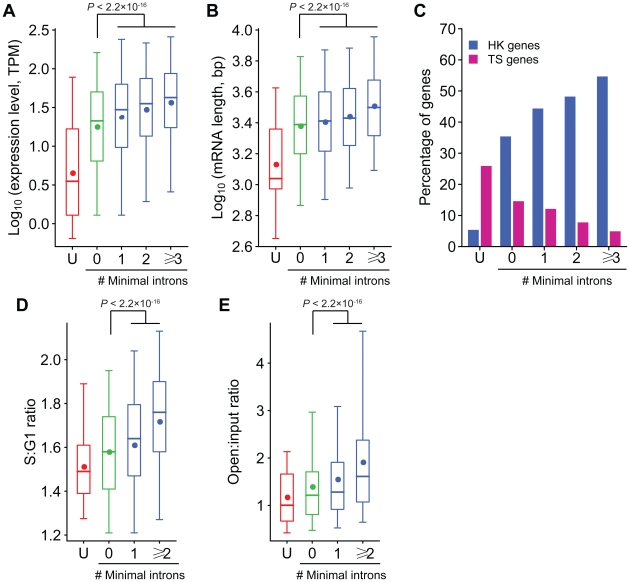
Characteristics of genes with minimal introns. Box-plot for mRNA level (A), mRNA length (B), replication time (D) and chromatin structure (E) and the fraction of housekeeping and tissue-specific genes (C) are shown for intron-less genes (boxes designated as “U”, red) and genes with different number of minimal introns. The boxes depict data between the 25th and 75th percentiles with central horizontal lines and solid circles representing the median and mean values, respectively, and whiskers showing 5th and 95th percentiles. *P*-values are calculated by the Wilcoxon rank sum test. Replicating time is indexed by S∶G1 ratio, with the larger value representing the earlier replication, and Chromatin status is demonstrated by open∶input ratio, with the larger value representing the more open chromatin structure [20], [21]. Due to the limited number of genes whose replication time and chromatin structure have been interrogated, the last two groups in panel D and E are pooled. Minimal intron-containing genes (blue boxes) tend to be universally expressed housekeeping genes with higher abundance and larger size, replicate earlier and have more open chromatin structure as compared to genes with only large introns (green boxes) and intron-less genes (red boxes).

In order to look further into the features of minimal introns-containing genes, we separated genes into three classes: (1) genes with minimal introns, (2) genes with only large introns and (3) genes without introns. We observed that intron-containing genes have significantly higher mRNA concentration and are significantly larger than genes without introns, consistent with the experimental observation that splicing can enhance mRNA nuclear export [10]. Moreover, the mRNA level and length are significantly greater in genes with minimal introns than in genes with only large introns (Wilcoxon rank sum test, *P*<2.2×10^−16^; Figure 4A and 4B). We previously showed that ubiquitously expressed housekeeping genes are in general highly expressed and have greater length in comparison with tissue-specific genes [24]. In consistence with this and above GO analysis, we observed housekeeping genes are significantly enriched in intron-containing genes and deficient in intron-less genes and the fraction of housekeeping genes is positively correlated with the number of minimal introns in intron-containing genes; the opposite trend was observed for tissue-specific genes (Figure 4C).

Comparing replication timing and chromatin structure among the three classes of genes, we found that the replication time of genes with minimal introns (with a mean S∶G1 ratio of 1.66) is significantly earlier (Wilcoxon rank sum test, *P*<2.2×10^−16^) than that of other spliced genes with only large introns (with a mean S∶G1 ratio of 1.58), which is again significantly earlier (Wilcoxon rank sum test, *P* = 4.1×10^−6^) than that of intron-less genes (with a mean S∶G1 ratio of 1.51; Figure 4D). The chromatin structure surrounding genes with minimal introns (with a mean open∶input ratio of 1.73) is significantly more open (Wilcoxon rank sum test, *P*<2.2×10^−16^) than that of other spliced genes with only large introns (with a mean open∶input ratio of 1.39), which is again significantly more open (Wilcoxon rank sum test, *P* = 4.6×10^−5^) than that of intron-less genes (with a mean open∶input ratio of 1.17; Figure 4E). Interestingly, the number of minimal introns within a gene has a quantitative nature, i.e., the more minimal introns a gene contains, the higher abundance, the larger size, the earlier replication timing and the more open chromatin structure it has.

## Discussion

**Figure 5:**
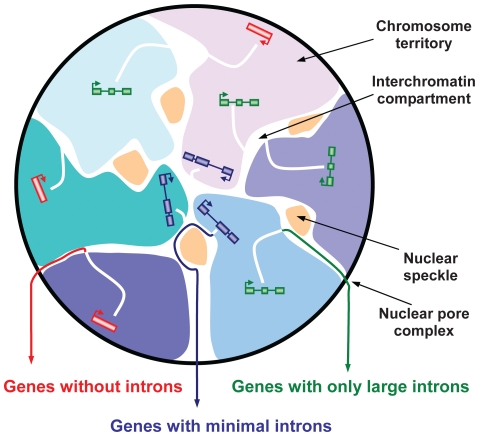
Nucleocytoplasmic export routing model. A schematic mammalian cell nucleus is depicted with nuclear structure annotated. Genes with minimal introns (blue) reside at the surface of chromosome territory lining interchromatin compartment space and/or near nuclear speckle to facilitate the unique splicing process. They generate large and highly abundant mRNPs that might be directly exported to cytosol. Genes with only large introns (green) and genes without introns (red) reside at interior locations and use different pathways to export. Cell use a routing system to selectively export the three sets of genes through somewhat independent pathways, preventing the entanglement of mRNPs and maximizing the efficiency of nuclear export.

In this study, we demonstrated that minimal introns are evolutionarily conserved and non-randomly distributed within genes and among chromosomes, indicating minimal introns have biological function and are specifically conserved during evolution. In order to unveil the function of minimal introns, we comparatively analyzed three sets of genes: (1) genes with minimal introns, (2) genes with only large introns and (3) genes without introns, and found that the presence of minimal introns is associated with gene's length, expression level, replication timing and chromatin structure. Based on these observatons, we proposed a nucleocytoplasmic export routing model (Figure 5). Genes with minimal intron reside at the surface of chromatin territories and near nuclear speckles to facilitate a specific splicing process. They generate large and highly abundant mRNPs that are directly exported to the cytosol. Genes with only large introns and intron-less genes locate at interior locations and use distinctive pathways to export. Cells use this routing strategy to selectively export the three types of genes, preventing the entanglement of mRNPs and maximizing the efficiency of nuclear export.

There are several pieces of evidence supporting our model. Nuclear pore complex (NPC) contains 9-nm aqueous channels, through which small water-soluble molecules are moved back-and-forth through passive diffusion; the channel of NPC expands to about 25 nm when exporting macromolecules, such as protein and mature mRNP, in an energy-dependent way [11], [25]. The nuclear transport is a highly orchestrated and rapid event; at least 10 molecules may traverse a NPC simultaneously [26]. At these busy and crowded “gates”, specific regulation is necessary for bulky and frequent objects. Striking examples are the insect Balbiani Ring (BR) genes that contain four small introns and generate mRNAs of more than 30 kb even after splicing. The exceptionally large and highly abundant BR mRNAs form giant mRNP particles that are folded into a compact ring-like structure with a diameter of 50 nm in nucleus. When exporting through the NPC, BR mRNP, one at a time, undergoes a series of structural unfolding and moves through as a thin fibril with 5′ end in the lead [25]. This regulated unfolding of giant BR mRNPs before nuclear export may have evolved to be a specific feature for highly abundant large genes. In this study, we showed that genes with minimal introns tend to be universally expressed housekeeping genes with higher cytoplasmic mRNA concentration and of larger size as compared to genes with only large introns and intron-less genes. These observations implied that during evolution, minimal introns may have been conserved in these highly abundant and large mRNAs to facilitate specific regulation on nucleocytoplasmic export.

How minimal introns specifically regulate the export of these highly abundant and giant mRNAs? In lower eukaryotes, introns are generally small and could be directly recognized by the splicing machinery—the intron definition model; but in higher eukaryotes, such as vertebrates where most of introns have been expanded, the splicing machinery would use exons as the unit of recognition to facilitate the identification of exons among the intronic oceans—the exon definition model [27], [28]. The evolutionarily conserved minimal introns may preserve the ancient intron-recognition pathway, while the large introns in vertebrates have been evolved to use the exon-recognition pathway. It is now known that spliceosome positions exon junction complexes (EJC) at 20–24 nt upstream of exon-exon junctions when introns are spliced out. It's reasonable to speculate that the two entirely different splicing mechanisms may locate different EJC at the splicing junction. At present, whether EJC is deposited at every exon-exon junction in mRNA with multiple introns and the full composition of EJC at every exon-exon junction remain to be elucidated [9]; but it is believed that the composition of EJC imprints mRNA with information required for many following steps of mRNA metabolism such as nucleocytoplasmic export, and these downstream processes are highly dependent on EJC position along the mRNA [9], [12]. Our observation that minimal introns are preferentially located toward 3′ end of mRNA is consistent with this position-dependent effect. We proposed that intron-specific alterations in EJC component, depending on intron position and intron length, may be an important variable. EJC at exon-exon junction surrounding minimal intron may have unique composition, imprinting minimal intron-containing genes with distinctive signals that guide a specific nucleocytoplasmic export pathway.

The current mammalian nuclear architecture model, i.e., chromosome territory - interchromatin compartment (CT-IC) model, depicts that chromosomes in the nucleus are organized as chromosome territories (CTs), which are sponge-like structure built up from condensed high-order chromatin fibers. CTs occupy spatially limited volume and are non-randomly positioned within the nucleus [19], [29], [30]. Although the molecular mechanisms that establish and maintain the nuclear architecture remain to be elucidated, the radial position of chromosomes has been related to some chromosome features such as gene density, chromosome size, replication time and transcriptional activity [31], [32], [33]. Recently, it was proposed that the non-random position of CTs is established in a self-organized way, i.e., the morphological appearance and spatial organization of CTs are determined by the sum of all functional properties of individual chromosome such as distribution of replication and transcriptional activity [6], [34]. We showed that minimal intron enrichment of chromosome is also a correlated feature. Therefore, the presence of minimal intron may also be involved in shaping the nuclear architecture, although the exact molecular mechanism needs further experiment to testify.

Interchromatin compartment (IC) is a contiguous network of DNA-free space, starting at nuclear pores and expanding between CTs and into their interior. IC can form lacunas with diameters of up to several micrometers containing nuclear bodies such as speckle, Cajal and PML bodies [19], [29], [30]. Loose/open chromatins reside at the surface of CT and expand into the IC, whereas the dense/compact chromatins are buried in CT interior. Early-replicating chromatins tend to locate at nuclear interior, whereas late-replicating chromatins locate at nuclear peripheral. We showed that genes without introns, genes with only large introns and genes with minimal introns have increasingly earlier replication time and more open chromatin structure. According to the present CT-IC model, the three classes of genes would locate at different nuclear position: genes with minimal introns would reside at CT surface and/or near the speckle domain, while genes with only large introns are relatively interior and genes without intron are deeply buried within CTs (Figure 5). Therefore, genes with minimal introns would undergo a unique splicing process and be directly exported to the nuclear pores through an independent pathway, preventing the entanglement of mRNPs produced in the interior of CTs.

So far, we still only have a very fragmented view of the molecular mechanism associated minimal intron. As the EJC composition can influence almost every stage of RNA metabolism, including export, localization, translation and NMD pathway [9], [35], in this study, although we proposed that the most likely role of minimal introns is to regulate nuclear export, we cannot rule out other possible involvement of minimal intron at current stage. Better knowledge of the detailed function of minimal introns entails further experiments. For example, directly visualizing nuclear spatial distribution of minimal intron by using interphase fluorescent *in situ* hybridization (FISH) and establishing mutated genes where minimal introns are artificially lengthened and/or disrupted would provide very important evidence for their function. Although the results presented in this study are largely descriptive and correlative and the model are hypothetical, these observations provided a necessary framework to design experiments to test the exact role of minimal introns in post-transcriptional regulation.

## Materials and Methods

We aligned 25,127 human and 21,153 mouse RefSeq transcripts (NCBI, March 13, 2008 update) onto their reference genomes (UCSC, hg18 and mm9) using BLAT program [36], and clustered them into 18,468 human and 18,889 mouse loci based on splicing-site-sharing for multi-exon transcripts and exon-overlapping for single-exon transcripts. When a locus has multiple alternatively spliced transcripts, the one with the greatest number of exons and/or length was selected as the representative. Refseq transcripts of *G. gallus*, *X. tropicalis* and *D. rerio* are processed in the same way. Intron positions are derived from RefSeq alignments. Human EST sequences and their genomic alignments were retrieved from UCSC annotation database (March 11, 2007 update), and clustered into RefSeq loci as previously described [37]. The number of ESTs associated with a RefSeq locus was used to index expression level; only non-normalized EST libraries with at least 100 ESTs were used for tag counting. The original sampling counts were converted into TPM (transcripts per million) for convenience. Expression breadth estimation across 18 human tissues was retrieved from previous study [37]; genes expressed in at least 16 of 18 tissues are defined as housekeeping genes and those expressed in at most 3 tissues as tissue-specific genes.

We retrieved 13,382 human-mouse orthologs from NCBI HomoloGene (Build 61), and aligned amino acid sequences by using CLUSTALW2 [38]. Ka/Ks ratios were calculated using CODEML program in PAML package [39]. Introns from orthologous genes and with the same position relative to the two coding sequences were defined as orthologous introns. 74,615 orthologous introns that have at least 3 flanking amino acids exactly aligned on each side were considered as highly reliable and were used for analysis in this study. For each human minimal intron, we randomly selected a position in large introns and intergenic regions and cut a stretch of sequence of equal length as control. The alignments of intronic and intergenic sequences were obtained according to the best chain alignment between human and mouse genomes from UCSC annotation database (August 15, 2007 update). The number of substitutions per site was calculated using BASEML program in PAML package.

Information on the replication time and chromatin structure of human genome were obtained from previous studies [20], [21], where DNA from S/G1 phase and open/compact chromatin were co-hybridized to a BAC clone array. Replicating time is measured by S∶G1 ratio, with the larger value representing the earlier replication; chromatin status is measured by open∶input ratio, with the larger value representing the more open chromatin structure. We retrieved 2,955 BAC clones from the array and mapped them onto the human genome hg18 by using UCSC precomputed coordinates (clonePos.txt, November 22, 2006 update). We obtained 2,683 non-overlapping clones with, on average, length of 150 Kb and spaced by 1 Mb. For each chromosome, the parameters are averaged among clones and weighted by the clone lengths. These clones interrogate 3768 loci which have similar fractions of minimal introns and intron length distribution as the total (data not shown). For each locus, the parameters of the overlapped clones were assigned.

## Supporting Information

Table S1 List of 6,327 human genes with minimal introns.
(1.30 MB XLS)

Figure S1 Gene ontology annotation of human genes with minimal introns.
(0.12 MB PDF)
