# Supplementary material for: A Novel Role for Minimal Introns: Routing mRNAs to the Cytosol
Source: PLoS One. 2010 Apr 12;5(4):e10144. doi: 10.1371/journal.pone.0010144 (PMC2856156; doi:10.1371/journal.pone.0010144)
Supplement: Figure S1 — Gene ontology annotation of human genes with minimal introns. (0.12 MB PDF) [file pone.0010144.s002.pdf]

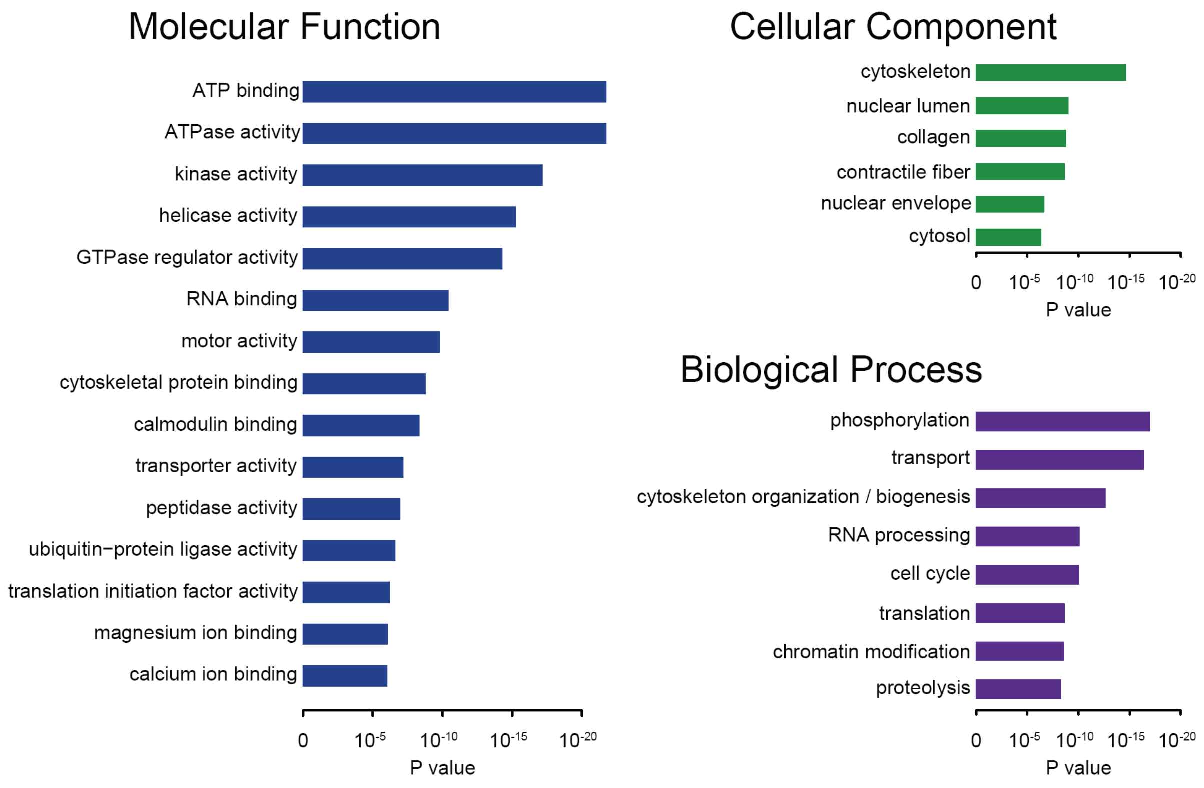

Figure S1. GO annotation of genes with minimal introns

The functional categories of genes with minimal introns are based on Gene Ontology (GO) annotations. Enriched functional terms are shown with *P*-values calculated according to the hypergeometric distribution.
